# Delayed surgery versus nonoperative treatment for hip fractures in post-COVID-19 situation

**DOI:** 10.1080/17453674.2020.1831242

**Published:** 2020-10-13

**Authors:** Viroj Wiwanitkit, Guohui Liu

**Affiliations:** Honorary professor, Dr DY Patil University, Pune, India; adjunct professor, Joseph Ayobaalola University, Ikeji-Arakeji, Nigeria; Department of Orthopedics, Union Hospital, Tongji Medical College, Huazhong University of Science and Technology, Wuhan, China

*Sir,—*I thank Viroj Wiwanitkit for his comments. There is a consensus that patients with hip fractures should be operated on without delay under ordinary circumstances. However, the outbreak of COVID-19 in Wuhan, China caused a large number of infected patients, who occupied a large amount of medical resources resulting in some hip fracture patients being unable to gain timely admission and surgery. Furthermore, some patients with hip fractures chose to stay at home, worried about being infected with COVID-19 in hospital. After the post-outbreak period several hip fracture patients began to visit hospitals seeking surgical treatment, but some patients still chose nonoperative treatment. We will continue to follow up these patients and provide them with free individual rehabilitation programs.

*Sir,—*I would like to comment on the publication “Delayed surgery versus nonoperative treatment for hip fractures in post-COVID-19 arena: a retrospective study of 145 patients [Mi et al. 2020].” Mi et al. concluded that “In hip fracture patients, delayed surgery compared with nonoperative therapy significantly improved hip function and reduced various major complications.” The result from this study is to be expected, as surgical management is generally recommended for treatment of hip fractures. The important consideration in this retrospective study is the reason for selection of a therapeutic alternative in each patient. However, the analysis of Mi et al. is in a “post-COVID-19 arena,” hence, there should be no reason for delayed surgery or nonoperative treatment of hip fractures.
